# A Word to Hospital Patients

**Published:** 1887-04-16

**Authors:** 


### A Word to Hospital Patients.

By One of Themselves.

The first word shall be, "Be patient." "Ah!" says some one, "that is easier said than done." Granted, my friend, but it can be done, nevertheless; and, after all, it's not so very difficult if you only go the right way about it. Make the effort, if you are inclined to be impatient, and you will be amply repaid. Anyone walking round a hospital ward, and taking note of the faces of its occupants, could almost predict the result of the various cases. Look, for instance, at that woman in bed No. 6. Look at her gloomy face, drawn and puckered and worried. What chance has physician or nurse in trying to cure her? No one was ever so bad as she is. She knows the doctor thinks very badly of her case. She heard him whispering something about her to the nurse, and she is quite sure that she will die—and so very likely she will, from sheer fretting and worrying. But now see that patient yonder. She is quite as bad, perhaps even worse, but she is calm and cheerful. When the pain comes she bears it quietly and bravely; when she is free from pain she looks at the bright side; speaks cheerfully; smiles brightly. As far as her strength goes, she finds some occupation, either for herself or others, and so the days go by, and the disease is gradually giving way to the treatment which has a fair field, until it is conquered, and the happy and thankful mother returns to her home and home duties once more. Away yonder in one of the gloomy dungeons of the Tower of London is a stone, on which words of wisdom were cut by a prisoner who suffered there over 300 years ago. The words are these: "The most unhappy man in the world is he who is not patient in adversity, for men are not killed by the troubles they have to bear, but by the impatience with which they bear them."

So far we have looked at only one aspect of patience, but now just consider what a wonderful difference it makes in the sufferer's relations to others. What a difference to the doctors, who give deep and earnest thought, sometimes most anxious thought, to the case in hand—what a difference, I say, it makes to them if they read patient endurance and hopeful courage in the face of their charge, or if that face shows only gloomy forebodings. What a difference also to nurses. They have much anxiety; much weary watching. Why should they be further tried by peevish fretfulness on the part of those they are doing their best to help? And then the poor fellow-sufferers in the world. Oh! what a weary time of it they have if one of these faint-hearted, miserable creatures gets amongst them. Oh, my friends, for your own sake, and for the sake of everyone about you, I say, "Be patient."

My next word shall be this—*Obedience*. Be obedient. You know, when sickness overtakes us it very soon puts us back again to childhood's helplessness, and with this helplessness comes childhood's duties. Now, one of the most important of these is obedience, and so I reiterate my injunction, be obedient. "Oh, nurse, that medicine is so nasty, I will not take it this afternoon." "Ah! but Dr. Smith said 'three times a day'; be obedient." "Nurse, I'm sure it can't matter about that rubbing to-night." "Twice a day; be obedient." "Nurse, I want beef-tea for supper." "Doctor said milk; be obedient." Now, this implicit obedience may seem a very trifling matter, but only the doctor can fully estimate its importance; and surely he has a right to expect that his injunctions and instructions shall be carried out in every detail.

And now just one other word. Be kind and helpful to your fellow-sufferers, more especially to new patients. The writer has spent many months as a patient in two large hospitals in London, and she made it a regular practice to speak a kindly word at the very first opportunity to each new comer—just a word or two of cheering hope and a gentle pressure of the hand, and the eye would brighten and the look of fear and dread would pass away, as the new patient recognised the fact that, perhaps, the dreaded hospital was not so bad after all. There are so many ways in which this helpful spirit may show itself. Are you well enough to move about the ward? Then spare a few minutes to sit beside one who is unable to get up, and, tired of her own thoughts, would be only too glad of a little gentle chat, or perhaps listening to a page or two of a pleasant book. Are you handy in the use of needle and thread? then help that poor woman yonder, whose wardrobe is of the scantiest, and whose special trouble just now is an ugly rent, which is beyond her powers of mending properly. There is one thing I would especially urge on patients, and that is, avoid all unkind criticism of each other, and more especially of the nurses and officials of the hospital. In all probability there will be many things you would wish otherwise: it is always so, wherever we may be; but such criticism never does good; on the contrary, it always does a great deal of harm. If there is anything you feel it a duty to notice, then speak directly, and with all Christian kindness, to the offending party on the subject, and on no account mention it to anyone else. There is another point on which I would urge all patients to be very firm, and that is to repress at once any coarse or immodest modes of expression. It will sometimes happen that a patient will come from surroundings which are not favourable to the culture of refined thought, but far otherwise. In such a case she will probably indulge in modes of expression which, to say the least, are objectionable—not because she is so very much worse than other people, but from simple force of habit. In such a case a kind word may do much, or even a discreet silence or a grieved look. Never join in the laugh which is raised at the expense of purity, and you will be surprised to find what a check you may be upon any such conduct. In closing, I would say, be watchful—watchful to save trouble. Do you want anything? Watch your opportunity to ask for it, so as to give as little trouble as possible. Be watchful, too, of the comfort of others. Your next neighbour, perhaps, has a headache, made worse to bear by the chatter which is going on around her. She will not speak for herself, but you can pass the word which will ensure her a quiet sleep and rest for the weary brain.

